# 

**Published:** 1871-03

**Authors:** 


### CONTENTS.

COMMUNICATIONS.

Investigations of the Tooth Pulp ... 97
Office Notes ... 104
Puffery ... 105
Gas *vs.* Chloroform ... 106
Ability *vs* Compensation ... 108
"Are the Mineral Acids formed in the Mouth?" ... 111
Associative Effort ... 114
Splitting Teeth with the Mallet ... 116
A Peculiar Case ... 117

PROCEEDINGS OF SOCIETIES.

New Jersey State Dental Society ... 119
Minutes of the Fifth Annual Meeting of the Ohio State Dental Society, Columbus, O., Dec. 6, 1870 ... 122

SELECTIONS.

A Cure for Love Melancholy ... 118
Influence of Diet on the Composition of Bone ... 118
Medical Education ... 130
Offensive Breath ... 132
Lancing the Gums ... 134
Styptic Wool ... 137
The Philosophy of Chills and Fever ... 138
The Discrimination between Syphilis and Scrofula ... 138
A Reversion Spectroscope ... 139
A War Victim ... 139
Hereditary Diseases ... 139

EDITORIAL.

Mistaken Impressions on Nitrous Oxyd ... 140

